# Dental invasive procedures in von Willebrand disease outpatients treated with high purity FVIII/VWF complex concentrate (Fanhdi®): experience of a single center

**DOI:** 10.1016/j.heliyon.2020.e03426

**Published:** 2020-02-25

**Authors:** Valeria De Padua, Umberto Romeo, Cristina Santoro, Riccardo Bosco, Erminia Baldacci, Antonietta Ferretti, Francesco Malaspina, Maria Gabriella Mazzucconi, Domenico Gaglioti

**Affiliations:** a Department Head-Neck, Sapienza University, Rome, Italy; b Department of Cellular Biotechnology and Hematology, Sapienza University, Rome, Italy

**Keywords:** Dentistry, Surgery, Dental materials, Dental surgery, Oral medicine, Periodontics, Prosthetic dentistry, Coagulation factor VIII, Oral surgery, FVIII/VWF concentrate, Postoperative bleeding, von Willebrand disease, von Willebrand factor

## Abstract

**Purpose:**

To retrospectively assess the effectiveness and safety of customized hemostatic protocols using a plasma-derived, von Willebrand Factor (VWF)-containing Factor VIII concentrate (pdVWF/FVIII) in von Willebrand disease (VWD) patients undergoing dental invasive procedures.

**Methods:**

Protocol for each patient was drawn up by the Blood Unit based on the VWD type, disease severity, and type of treatment. pdFVIII/VWF infusions and doses were registered at 30–60 min before intervention (t0) and at 12-24-36-48-72 h after intervention (t12-t72) and up to day 7. Any peri- or postoperative bleeding, complication or adverse event was registered.

**Results:**

Forty-five dental procedures were performed on 20 VWD patients (six type-1, two type-2a, six type-2b, six type-3). Most pdFVIII/VWF infusions at t0 were 60 IU/kg (n = 7) and 50 IU/kg (n = 9). Subsequent infusions were mostly 30–50 IU/kg. No bleeding complications or adverse events were reported.

**Conclusion:**

This study supports the safety and efficacy of pdFVIII/VWF to prevent peri- and postoperative bleeding after invasive oral procedures.

## 1 Introduction

Von Willebrand disease (VWD) is a hereditary hemorrhagic disorder caused by a genetic defect with a mutation of the gene encoding the von Willebrand Factor (VWF), located on chromosome 12, which causes a quantitative, structural or functional fault, in the VWF [1]. It occurs with a delay in the formation of the platelet plug, due to the altered adhesion of platelets to the subendothelium [2].

Around 26% of the population with hemostasis disorders suffers from it and it affects men and women in the same proportions [3]. There are three VWD forms: type 1 and type 3 with the partial and total quantitative defect of the VWF, respectively, and type 2 with the qualitative defect of the VWF. Type 1 VWD patients accounts for up to 70% of all VWD cases whereas type 2 and 3, 20% and 5% respectively. Sadler separates type 2 into 4 variants (2A, 2B, 2M, 2N) according to some very precise phenotype characteristics: type 2A, with a VWF-dependent platelet adhesion defect because of the decrease of the higher molecular weight multimers of VWF; type 2B, caused by a pathological increase of the platelet-VWF aggregation consequent to a fault at the level of the binding site; type 2M with marked reduction of the VWF activity, not due to a lack of high molecular weight multimers; and type 2N, with a greater reduction in the level of Factor VIII (FVIII) compared to the VWF [1, 4].

In patients with VWD, bleeding is the adverse event that can happen during invasive procedures [5, 6]. In invasive oral surgery, bleeding can be controlled by different therapeutic approaches. Desmopressin (DDAVP) is used in cases where there is a positive response to the test, in patients with type 1 VWD with basic levels of FVIII and VWF >10 U/dL [7, 8], while in other VWD subtypes the response to DDAVP is significantly reduced. Response to DDAVP is, nevertheless, active in types 2A and 2M, while in type 2B it is contraindicated due to the onset or transient aggravation of thrombocytopenia [7, 9, 10]. DDAVP is ineffective in VWD types 3 and 2N where plasma-derived VWF-containing Factor VIII concentrates (pdFVIII/VWF) are used [11, 12].

This retrospective study was aimed to highlight the effectiveness of customized therapeutic protocols with a highly purified pdVWF/FVIII administered on patients after invasive oral procedures, in association with local hemostatic control measures, in order to reduce the risk of peri- and postoperative bleeding.

## 2 Materials and methods

This study was conducted in accordance with the ethical principles that have their origins in the Declaration of Helsinki and approved by Ethics Committees of the respective sites if required by local rules.

VWD type and subtype were diagnosed by hematologists specialized in hemostasis and thrombosis at Sapienza University, in accordance with the recommendations of the VWF Scientific Sub-Committee of the International Society of Thrombosis and Haemostasis, as published [4, 13]. The medical records were examined between June 2007 and April 2019. Patients with VWD who presented oral diseases were referred to the Stomatology Unit of Sapienza University for a first clinical and radiographic examination. Those patients who underwent oral surgery requiring pdVWF/FVIII infusion according to a tailored therapeutic protocol decided by the hematologists were included in the study.

Primary endpoint of the study was to assess if customized therapeutic protocols with a highly purified pdVWF/FVIII prevented peri- and postoperative bleeding in patients with VWD who were subjected to invasive oral procedures. Safety assessment was the secondary endpoint. Inflammatory complications as well as any other adverse event (AE) during the administration of the substitution factors were recorded. The AEs were defined as unexpected medical side-effects. All analyses performed were descriptive.

The hematological protocol planned the infusion of highly purified pdFVIII/VWF concentrates in all patients examined. The pdFVIII/VWF infusion protocol customized for each patient was drawn up by the Blood Establishment based on the type of VWD, on the degree of severity, and on the type of intervention. The pdFVIII/VWF concentrate used was Fanhdi® (Grifols, Barcelona, Spain). Infusions of pdFVIII/VWF concentrate and doses (IU/kg) were registered at t0 (30 min–1 h before the intervention) as well as at t12, t24, t36, t48 t72 (12, 24, 36, 48 and 72 h after intervention) and up to day 7.

Local anesthesia by infiltration with mepivacaine and vasoconstrictor (Mepivacaina Pierrel; Pierrel Pharma Srl, Capua CE, IT) was administered nearby surgical site, while loco-regional anesthesia with mepivacaine without vasoconstrictor was administered for the inferior alveolar nerve block. In cases of dental extraction, we proceeded to plug with gelatin sponges (Gelita-spon®; Gelita Medical, Amsterdam, Holland) and application of thawed fibrin glue (Tissucol/Tisseel, Baxter, Deerfield IL, US) until bleeding stopped. The use of non-traumatic cylindrical needles with resorbable suture thread 4.0 (Vicryl; Ethicon Inc., Johnson & Johnson New Brunswick NJ, USA) were used from approximation. The surgical site was compressed with gauze soaked with tranexamic acid (Tranex, Malesci Spa, Grassina FI, Italy) for 15 min intermittently. Upon the completion of the local hemostasis protocol, synthetic ice was discontinuously applied externally for three hours.

In cases of subgingival scaling and root-planning, full mouth disinfection was carried out with chlorhexidine 0.20% based mouthwash (Corsodyl; Glaxosmithkline, Brentford, United Kingdom) for two minutes. Some patients were treated with a mucoperiosteal open flap scaling and root-planning, in association with antibiotic therapy for six days.

After the periodontal treatment, the patient was asked to keep 10 mL of tranexamic acid in patient's mouth for one to two min, to be swallowed subsequently.

In the excisional biopsy intervention, hemostasis was ensured by suture and gauze compression with tranexamic acid. Home rinses with tranexamic acid were prescribed in the three days after the intervention, three times daily, for each surgical procedure.

Paracetamol was the drug of choice for the analgesic therapy, with prohibition of using NSAIDs. Postoperative personalized follow-up was performed in the days following the intervention, to examine any bleeding, inflammatory reactions and pain control. The patient was given instructions about nutrition and oral hygiene at home.

## 3 Results

Twenty patients suffering from VWD (nine males, eleven females) with a median age of 52 years (range 15–86) were treated: six type 1, two type 2a, six type 2b, and six type 3. Details for each patient are reported in Table 1.

**Table 1:** Patients’ von Willebrand disease (VWD) profile and characteristics of the dental and hemostatic procedures.

VWD type	Patient no.	Laboratory parameters				pdFVIII/VWF dosage (IU/kg/day)							Surgical intervention
		FVIII:C (%)	VWF:Ag (%)	VWF:Rco (%)	RIPA (mg/mL)	t0	t12	t24	t36	t48	t72	Further	
1	1	53	40	28		60	30	30	30	30		30, for two more days	Three scaling and roots planning; one incisional biopsy
	2	29	7	6		60	50	50		50		30, until day 7 after intervention	Three dental extractions
	3	30	30	25		60	30	30	30	30	30	30, until day 7 after intervention	Two dental extractions
	4	37	8	10		60	30	30	30	30		30, for three more days	Two dental extractions
	5	22	10	9		50	25	25					Wisdom tooth extraction
	6	68	75	50	0.12	30						Tranexamic acid, vial 500 mg orally every 6 h until day 7 after intervention	One scaling and root planning; one dental extraction; one excisional biopsy
2a	7	32	27	8	1.32	40	40	40	40	40		20, alternate until day 7 after intervention	Abscess drainage
	8	53	23	6	1.32	50	30	30		30	30	Tranexamic acid, 500 mg 3 tablets/day orally until day 7 after intervention	Wisdom tooth extraction
2b	9	28	40	25	0.18	50	30	25	25	25	25	25, until day 7 after intervention	Five dental extractions
	10	33	46	24	0.4	40	40	40		40	40	30, day 4	One impacted wisdom tooth surgical extraction
	11	60	58	30	0.2	50		50					One dental extraction
	12	35	49	8	1.09	30							Scaling and root planning
	13	28	23	10	0.6	60	30	30	30			30, until day 7	One dental extraction; one scaling and root planning
	14	30	42	10	1.48	30							One deciduous dental extraction
3	15	7	3.7	<6.25		50	30	30		30		Tranexamic acid, 500 mg 3 tablets/day orally until day 7 after intervention	Two impacted wisdom tooth surgical extractions
	16	1.5	1	<6.25		60	50	50		50		50, until day 7 after intervention	Five dental extractions; one impacted wisdom tooth surgical extraction
	17	3	2.5	<6.25		50	30	30		30		30, until day 7 after intervention	One dental extraction, one wisdom tooth extraction
	18	1.68	2.1	<6.25		60	50	50	30	30	50	50, until day 7 after intervention	Three dental extractions; one impacted wisdom tooth dental extraction; one scaling and root planning
	19	7	3	<6.25		50		50					One scaling and root planning
	20	3	2.5	<6.25		60	50	50		50		50, until day 7 after intervention	One dental extraction

FVIII:C: Factor VIII procoagulant activity; VWF: von Willebrand factor; pdFVIII/VWF: plasma-derived VWF-containing FVIII concentrate; RIPA: ristocetin-induced platelet agglutination; VWF:Ag: von Willebrand factor antigen; VWF:Rco: ristocetin cofactor activity in plasma.

Forty-five dental procedures were performed, meaning each procedure performed in a single surgical session. At least one infusion of pdFVIII/VWF concentrate was performed in all patients at t0 and the other infusions were decided on the bases of laboratory parameters, clinical bleeding history and type of surgery. All procedures were considered invasive because they involved manipulation of the gums or perforation of the oral mucosa.

Twenty-six dental extractions, eight wisdom tooth extractions (5 impacted), two biopsies, eight scaling and root-planning and one abscess drainage were performed, without peri- or postoperative bleeding. Table 1 specifies the types of intervention and therapeutic infusion protocols customized for each patient. Most infusions at t0 were 60 IU/kg (n = 7) and 50 IU/kg (n = 7). Subsequent infusions up to day 7 were mostly 30–50 IU/kg (Table 1).

For the primary endpoint, there were no peri- or postoperative bleeding, and secondary inflammatory complications. All patients under examination diligently followed the postoperative hematologic protocol. Patients enabled and authorized to self-infuse continued the substitution therapy independently at home. Patients not authorized to self-infuse underwent the treatment at the Blood Unit. No adverse events were recorded during the administration of the substitution factors.

## 4 Discussion

Any invasive dental treatment exposes patients with hemostasis disorders to a high risk of peri- and postoperative bleeding [11, 14, 15]. In our study, 20 patients suffering from VWD underwent oral procedures with pdFVIII/VWF concentrate prophylaxis, without any bleeding problems.

In the literature, the incidence of postoperative bleeding after invasive dental treatments in patients with appropriate hemostasis is estimated at between 0.2% and 3.3%, while the occurrence of postoperative bleeding after oral surgery in patients with hemostasis disorders is in the range 8.6%–32.1% [16, 17]. In comparison, the postoperative recovery of our patients infused with the pdFVIII/VWF was similar to that of patients not suffering from hemostasis disorders. The use of specific hematological protocols is therefore necessary to minimize the risk of hemorrhage [12, 15, 18].

The close collaboration between Blood Unit and Stomatology Unit is essential to operate on the patient safely [19, 20, 21]. The results obtained in this study, taking in account the small sample size (only patients treated with high purity FVIII/VWF complex concentrate were considered), and the data provided in the literature support the fact that the administration of pdFVIII/VWF concentrates, according to the specific surgical needs, is effective in controlling hemostasis [12, 18, 20].

In our protocol, local anesthesia allow reducing the risk of bleeding [3]. In addition, a scrupulous local hemostasis protocol with fibrin glue or gelatin sponges and resorbable suture ensured a good sealing of the wound and a postoperative recovery [22, 23]. In our center the use of resorbable suture is preferred since the removal of stitches would expose the patient to further trauma, with risk of bleeding [11, 24].

The protocols implemented in this study showed patients not to have bleeding difficulties postoperatively, because they have been instructed to continue the IV infusion of pdFVIII/VWF, and to rinse with tranexamic acid three times daily for three days after the intervention. The rinsing of the latter procedure guarantees a high concentration of tranexamic acid inside the oral cavity that limits the fibrinolytic action of the salivary enzymes [25]. Self-infusion is important to reduce hospitalization with consequent saving of economic resources and better patient compliance [26].

To summarize, peri- and postprocedural care for dental invasive procedures in VWD is an underexposed field in the literature. An active collaboration between Blood Unit and Dental Unit is necessary [26]. Despite the limitations of this study associated with its retrospective nature, small sample size and the lack of a control group, our results emphasize the importance of the implementation of appropriate custom hematological protocols combined with a local hemostasis protocols in the management of patients with VWD. These protocols avoid peri- and postoperative bleeding after invasive dental procedures. The administration of a highly purified pdFVIII/VWF concentrate was safe and effective.

## Declarations

### Author contribution statement

V. De Padua, A. Ferretti, C. Santoro, and D. Gaglioti: Conceived and designed the experiments; Performed the experiments; Analyzed and interpreted the data; Contributed reagents, materials, analysis tools or data; Wrote the paper.

R. Bosco and U. Romeo: Contributed reagents, materials, analysis tools or data.

E. Baldacci: Conceived and designed the experiments.

F. Malaspina: Analyzed and interpreted the data.

M. Mazzucconi: Performed the experiments.

### Funding statement

This research did not receive any specific grant from funding agencies in the public, commercial, or not-for-profit sectors.

### Competing interest statement

The authors declare no conflict of interest.

### Additional information

No additional information is available for this paper.
